# Plummer–Vinson syndrome in a 10‐year‐old boy from Côte d'Ivoire: An exceptional paediatric case with African context

**DOI:** 10.1002/jpr3.70227

**Published:** 2026-08-03

**Authors:** Paul Mike Tayou Mbobda, Hatrydt Guillaume Dimitri Kouamé, Amadou Ouattara

**Affiliations:** ^1^ Department of Medecin Loum District Hospital Loum Cameroon; ^2^ Service d'Hépato‐Gastro‐Entérologie, CHU de Yopougon Abidjan Côte d'Ivoire; ^3^ Faculté de Médecine, Département des Maladies de l'Appareil Digestif Université Félix Houphouët‐Boigny Abidjan Côte d'Ivoire; ^4^ Service d'Hépato‐Gastro‐Entérologie, CHU de Cocody Abidjan Côte d'Ivoire

**Keywords:** endoscopic dilation, iron‐deficiency anaemia, oesophageal web, paediatric endoscopy, sideropenic dysphagia

## Abstract

Plummer–Vinson syndrome (PVS) is characterised by the triad of dysphagia, iron‐deficiency anaemia, and proximal oesophageal webs. While well described in adults, paediatric cases remain exceptionally rare, particularly in sub‐Saharan Africa. We report a 10‐year‐old boy from Côte d'Ivoire, presenting with progressive dysphagia of 3 years' duration and severe iron‐deficiency anaemia (haemoglobin 6.0 g/dL) with neither haemoglobinopathy nor ova or parasites. Upper gastrointestinal endoscopy demonstrated a semi‐circumferential post‐cricoid oesophageal web; with normal surrounding mucosa and histology. Endoscopic balloon dilation combined with weight‐based oral iron supplementation co‐administered with ascorbic acid resulted in complete clinical and haematological recovery, sustained at 1‐year follow‐up. This case underscores the importance of recognising iron‐deficiency anaemia associated with dysphagia in children. Combined endoscopic dilation and appropriately dosed iron supplementation with ascorbic acid are effective. Increased awareness and access to endoscopic evaluation are essential to ensure timely diagnosis and effective management in low‐resource African settings.

## 1 INTRODUCTION

Plummer–Vinson syndrome (PVS), also known as Paterson–Brown–Kelly syndrome, is characterised by the association of dysphagia, iron‐deficiency anaemia, and proximal oesophageal webs. The condition predominantly affects middle‐aged women and is now rarely encountered in high‐income settings, likely due to improved nutritional status and early treatment of iron deficiency. In contrast, paediatric cases are exceptional, with only isolated reports worldwide, and documentation from sub‐Saharan Africa is exceedingly scarce.^1^, ^2^, ^3^, ^4^ The high regional prevalence of haemoglobinopathies and helminthic co‐infections in West Africa further necessitates a rigorous and systematic diagnostic approach when evaluating children with chronic microcytic anaemia and dysphagia.^5^, ^6^

Iron deficiency is considered central to the pathophysiology of PVS, leading to epithelial atrophy, impaired oesophageal motility, and subsequent web formation.^5^, ^6^ In regions where iron‐deficiency anaemia is highly prevalent, particularly in low‐resource African settings, PVS may be underrecognised due to limited access to endoscopy and overlapping nutritional disorders. We report a paediatric case of PVS from Côte d'Ivoire and discuss its clinical relevance in the context of published paediatric data.

## 2 CASE REPORT

A 10‐year‐old boy from Abidjan, Côte d'Ivoire, presented with progressive dysphagia to solid foods evolving over 3 years. The dysphagia was not associated with odynophagia, vomiting, weight loss, respiratory symptoms, or gastro‐oesophageal reflux. He had a history of recurrent severe anaemia with poor response to oral iron therapy. There was no history of caustic ingestion, prolonged intubation, chronic inflammatory disease, atopy, or blistering skin disorders.

**Figure 1:**
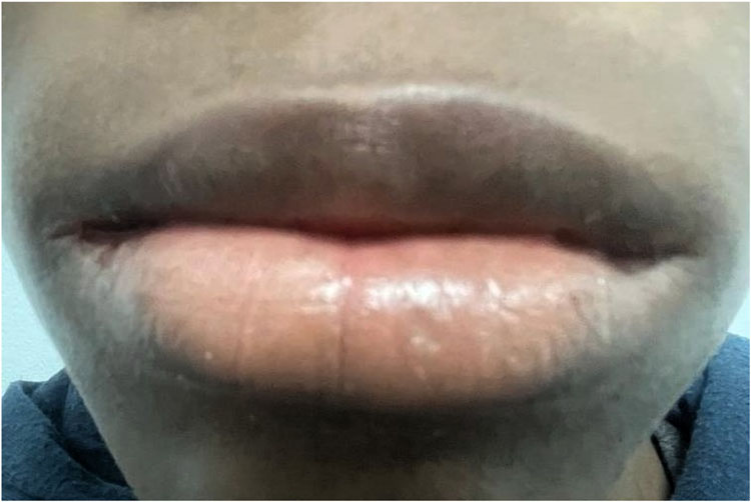
Angular chelitis.

**Figure 2:**
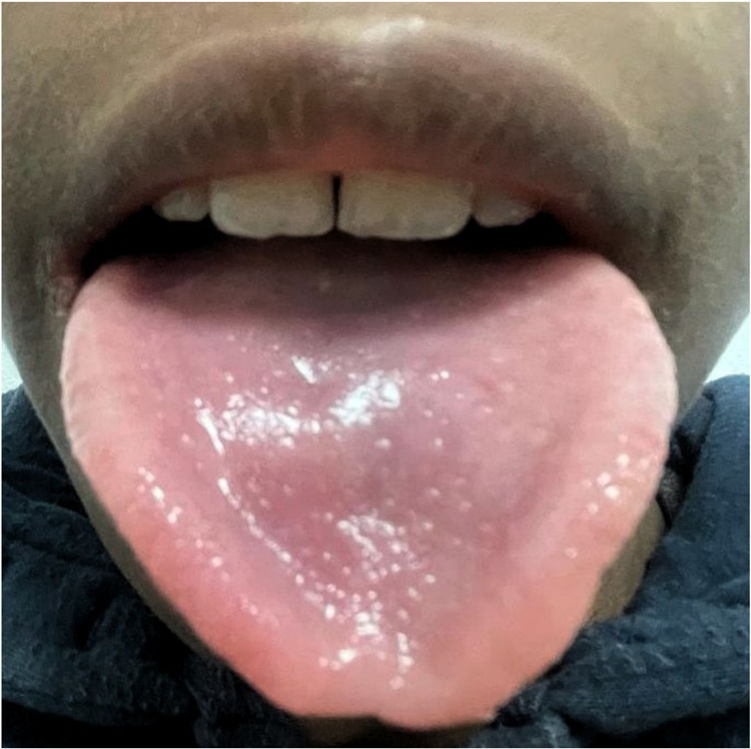
Atrophic glositis.

On examination, the child appeared pale with angular cheilitis (Figure 1), atrophic glossitis (Figure 2), and koilonychia. No lymphadenopathy or hepatosplenomegaly was detected. Weight and height were at the 10th percentile for age (body weight 30 kg).

Laboratory investigations revealed severe microcytic hypochromic anaemia with haemoglobin 6.0 g/dL, mean corpuscular volume 70 fL, red blood cell count 3.2 × 10^12^/L, ferritin 15 ng/mL, serum iron 40 μg/dL, transferrin saturation 17%, and elevated total iron‐binding capacity (500 μg/dL) with a Mentzer index (mean cell volume/red blood cell count) higher than 13, consistent with iron‐deficiency anaemia rather than thalassaemia trait. Peripheral blood smear demonstrated anisopoikilocytosis. Haemoglobin electrophoresis demonstrated a normal HbAA pattern, effectively excluding sickle cell disease, haemoglobin C disease, and beta‐thalassaemia. Stool microscopy for ova and parasites was negative. Inflammatory markers (C‐reactive protein and erythrocyte sedimentation rate) were not obtained at initial presentation; the implications of this are addressed in the Section 3.

It is noteworthy that prior oral iron supplementation had been prescribed on two separate occasions at a subtherapeutic dose (approximately 3 mg/kg/day of elemental iron) for 4‐week courses, without concomitant ascorbic acid supplementation. Suboptimal dosing, insufficient treatment duration, and the absence of a bioavailability enhancer most plausibly account for the apparent refractoriness to therapy documented prior to the current presentation.

**Figure 3:**
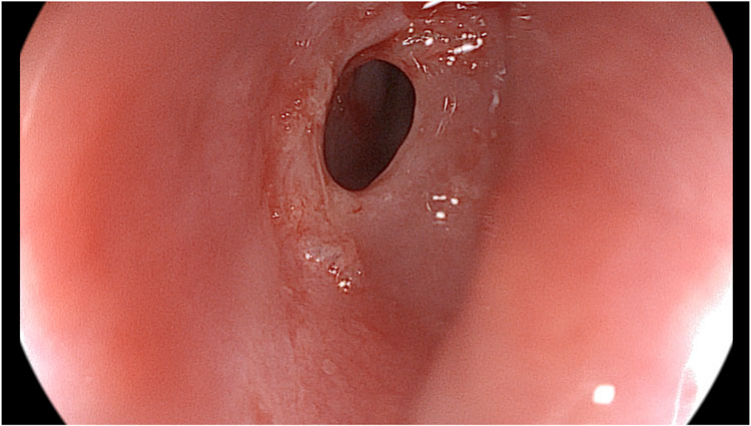
Endoscopic image of post‐cricoid oesophageal web.

Upper gastrointestinal endoscopy showed a thin, semi‐circumferential post‐cricoid oesophageal web located 15 cm from the dental arches, causing partial luminal obstruction (Figure 3). The surrounding mucosa appeared normal, and oesophageal biopsies revealed normal squamous epithelium. Alternative causes of chronic dysphagia and iron‐deficiency anaemia were excluded based on clinical, endoscopic, and histological findings.

Endoscopic balloon dilation was performed successfully. Oral ferrous sulphate was prescribed at a weight‐based dose of 6 mg/kg/day of elemental iron, combined with ascorbic acid 100 mg daily, for 3 months. Dysphagia resolved completely, and haematological parameters normalised within 8 months. At 1‐year follow‐up, the patient remained asymptomatic with no recurrence.

## 3 DISCUSSION

This case illustrates an exceptionally rare presentation of paediatric PVS in sub‐Saharan Africa. The absence of previously reported cases from West Africa likely reflects underdiagnosis rather than true rarity. Our patient exhibited the classical triad of dysphagia, iron‐deficiency anaemia, and a post‐cricoid oesophageal web, consistent with published paediatric reports.^1^, ^2^, ^3^, ^4^, ^7^

A synthesis of the available paediatric literature indicates that dysphagia is the universal presenting symptom, often evolving insidiously over months or years, and invariably associated with iron‐deficiency anaemia.^1^, ^2^, ^3^, ^4^, ^7^, ^8^, ^9^, ^10^ Laboratory findings consistently demonstrate microcytic hypochromic anaemia with low ferritin and transferrin saturation. Endoscopy remains the diagnostic gold standard, allowing direct visualisation of oesophageal webs and exclusion of alternative pathology, while contrast studies are adjunctive rather than essential.^4^, ^7^

Iron supplementation constitutes the cornerstone of therapy; however, dysphagia frequently persists despite haematological correction, necessitating endoscopic dilation. In our patient, apparent refractoriness to prior oral iron therapy is best explained by subtherapeutic dosing without concomitant ascorbic acid, underscoring the critical importance of prescribing weight‐based elemental iron at 3–6 mg/kg/day with vitamin C co‐supplementation to optimise intestinal absorption, particularly in resource‐limited settings where dietary iron intake may be chronically insufficient. Balloon or Savary–Gilliard dilation has been shown to be effective and safe in paediatric patients, with excellent long‐term outcomes and no reported major complications or recurrences.^1^, ^2^, ^3^, ^4^, ^10^ Our patient's favourable evolution aligns with these observations.

Although adult PVS is associated with an increased risk of post‐cricoid squamous cell carcinoma, no malignant transformation has been reported in paediatric cases.^6^ It should be noted, however, that follow‐up duration in most published paediatric series is insufficient to permit definitive conclusions regarding long‐term oncological risk. Accordingly, periodic endoscopic surveillance following treatment is advocated, though the optimal surveillance interval has yet to be established by prospective data.^9^ Early recognition and treatment remain essential to prevent nutritional compromise and long‐term morbidity. In African settings, improving awareness and access to diagnostic endoscopy is critical for timely diagnosis.

## 4 CONCLUSION

Paediatric PVS is rare but clinically recognisable. Progressive dysphagia associated with iron‐deficiency anaemia should prompt consideration of this diagnosis, even in low‐resource settings. A systematic diagnostic workup should include haemoglobin electrophoresis and parasitological evaluation, particularly in sub‐Saharan Africa, and oesophageal biopsies must document eosinophil counts per high‐power field to formally exclude eosinophilic oesophagitis. Combined iron supplementation at an appropriate weight‐based dose with concomitant ascorbic acid and endoscopic dilation are safe and effective. Increased awareness in sub‐Saharan Africa is essential to improve detection and outcomes.

## CONFLICT OF INTEREST STATEMENT

The authors declare no conflicts of interest.

## ETHICS STATEMENT

An informed consent for the publication of this case report, including any accompanying images, was obtained from the patient's legal guardian.
